# A Descriptive Catalogue of the Anatomical Museum of St. Bartholomew's Hospital. Vol. I, Containing the Description of the Specimens Illustrative of Pathological Anatomy

**Published:** 1847-01

**Authors:** 


ART. XII.—*A descriptive Catalogue of the Anatomical Museum of St. Bartholomew's Hospital. Vol. I, containing the description of the specimens illustrative of Pathological Anatomy.—London,* 1846. 8vo, pp. 487.

THIS is a catalogue of a very extensive and valuable collection, and is itself a model of what such catalogues should be. The specimens are arranged in series (thirty-eight in all), each series being preceded by classified tables of reference to the objects contained in it, by which means the advantages of an arrangement, founded on pathological principles, are attained, without interfering with the ordinary and convenient method of displaying preparations. "By the help of these tables it will be easy both to find any specimen in the museum, and to study the preparations in each series in the order in which they may best serve for illustrations of the disease of the part to which that series is devoted." There is also prefixed to the work "a general table of references to the principal specimens for the illustration of general pathology." This is a most valuable key to the student, as by means of it he can at once refer to any of the specimens, however dispersed over the museum, almost as conveniently as if they had been arranged in a separate series for the special purpose of illustrating general pathology. All these tables, and indeed the greater portion of the catalogue, are the work of Mr. Paget; and the whole bears the impress of his master hand.

